# Mapping essential somatic hypermutations in a CD4-binding site bNAb informs HIV-1 vaccine design

**DOI:** 10.1016/j.celrep.2025.115713

**Published:** 2025-05-15

**Authors:** Kim-Marie A. Dam, Harry B. Gristick, Yancheng E. Li, Zhi Yang, Priyanthi N.P. Gnanapragasam, Anthony P. West, Michael S. Seaman, Pamela J. Bjorkman

**Affiliations:** 1 Division of Biology and Biological Engineering, California Institute of Technology, Pasadena, CA 91125, USA; 2 Division of Chemistry and Chemical Engineering, California Institute of Technology, Pasadena, CA 91125, USA; 3 Center for Virology and Vaccine Research, Beth Israel Deaconess Medical Center, Harvard Medical School, Boston, MA 02215, USA

**Keywords:** HIV-1 broadly neutralizing antibodies, IOMA-class antibodies, somatic hypermutation, SHM, CD4-binding site, CD4bs, HIV vaccine design, germline-targeting immunogens, cryo-EM structure, neutralization breadth and potency, Env glycan accommodation

## Abstract

HIV-1 broadly neutralizing antibodies (bNAbs) targeting the CD4-binding site (CD4bs) contain rare features that pose challenges to elicit these bNAbs through vaccination. The IOMA class of CD4bs bNAbs includes fewer rare features and somatic hypermutations (SHMs) to achieve broad neutralization, thus presenting a potentially accessible pathway for vaccine-induced bNAb development. Here, we created a library of IOMA variants in which each SHM was individually reverted to the inferred germline counterpart to investigate the roles of SHMs in conferring IOMA’s neutralization potency and breadth. Impacts on neutralization for each variant were evaluated, and this information was used to design minimally mutated IOMA-class variants (IOMAmin) that incorporated the fewest SHMs required for achieving IOMA’s neutralization breadth. A cryoelectron microscopy (cryo-EM) structure of an IOMAmin variant bound to Env was used to further interpret characteristics of IOMA variants to elucidate how IOMA’s structural features correlate with its neutralization mechanism, informing the design of IOMA-targeting immunogens.

## Highlights

• IOMAmin variants with reduced SHMs retain neutralization potency and breadth
• Cryo-EM structure reveals that IOMAmin preserves key Env interactions despite fewer SHMs
• IOMA variants with mutations in CDRH3 and CDRL1 fail to improve neutralization

## Graphical abstract

**Figure:**
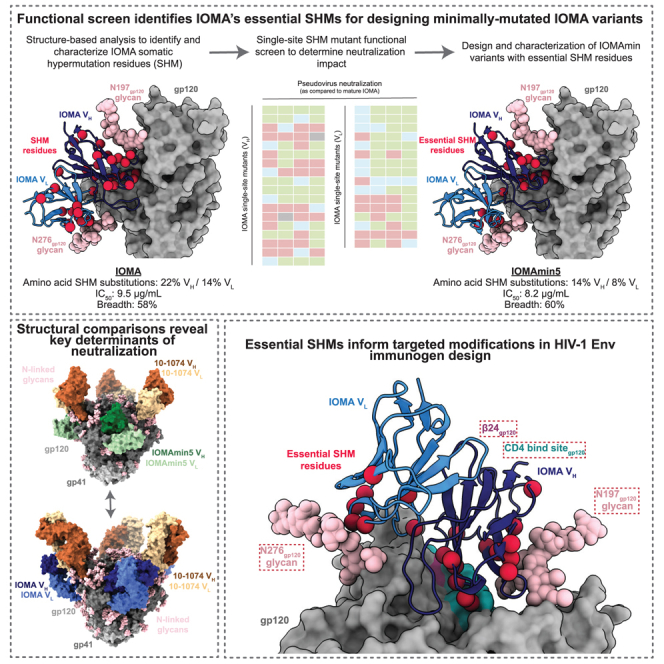

## Teaser

Dam et al. identify essential somatic hypermutations (SHMs) in the IOMA class of CD4-binding site broadly neutralizing antibodies to guide HIV-1 vaccine design. By systematically reverting SHMs, they define minimal mutations required for IOMA’s neutralization potency and breadth. Structural and functional analyses of IOMAmin variants highlight key determinants for immunogen development.

## Introduction

The discovery and extensive characterization of broadly neutralizing antibodies (bNAbs) against HIV-1 that potently neutralize a large fraction of circulating isolates have provided avenues to combat the ongoing HIV-1/AIDS pandemic, including the development of bNAbs for passive transfer and providing templates for vaccine design.^1^^,^^2^^,^^3^^,^^4^^,^^5^^,^^6^ bNAbs target several epitopes on envelope (Env), the sole viral glycoprotein on the surface of HIV-1, and 3D structures of Env-bNAb complexes have informed our understanding of bNAb features that confer diverse neutralization to enable structure-based immunogen design.^6^^,^^7^^,^^8^^,^^9^^,^^10^^,^^11^^,^^12^ Although substantial progress has been made on this front, an HIV-1 vaccine capable of eliciting bNAbs and providing robust and diverse protection has yet to be developed.

The binding site for CD4 (CD4bs), the HIV-1 host receptor, is an attractive target for HIV-1 immunogen design because bNAbs that recognize this epitope are among the most potent and broad.^6^^,^^12^^,^^13^^,^^14^^,^^15^^,^^16^^,^^17^ Many of these bNAbs share distinct variable heavy (V_H_) gene segments that encode particular features compatible with the CD4bs epitope and display impressive neutralization potencies.^12^^,^^14^^,^^18^ One class of CD4bs bNAbs that is V_H_1-2 gene segment restricted includes two sub-classes: the well-studied VRC01 class^9^^,^^13^^,^^14^^,^^16^^,^^18^^,^^19^^,^^20^^,^^21^ and the more recently described IOMA class.^8^^,^^22^ Although these bNAb sub-classes share a V_H_ gene segment ontogeny, they have distinct sequence characteristics and binding mechanisms. The VRC01 class is defined by its rare, five-residue light-chain complementarity-determining region (CDRL3) that is present in less than 1% of human antibody light-chain genes.^13^^,^^18^ Other features include generally high levels of somatic hypermutation (SHM) and a CDRL1 with deletions or multiple glycine mutations necessary to accommodate the N276_gp120_ glycan.^13^^,^^18^ In contrast, the IOMA class of CD4bs bNAbs is characterized by an 8-residue CDRL3, which is more commonly represented in the human B cell repertoire compared to VRC01 class five-residue CDRL3s.^8^^,^^22^ Additionally, IOMA-class antibodies only require a single glycine mutation in CDRL1 to accommodate the N276_gp120_ glycan, a feature that is easier to achieve through vaccination than the VRC01-class CDRL1 changes. Finally, IOMA-class bNAbs have lower levels of SHM compared to the majority of bNAbs within the VRC01 class.^8^^,^^22^ High levels of SHM associated with HIV-1 CD4bs bNAbs are considered a hurdle in vaccine development,^10^^,^^23^ as these mutations often require several years to accumulate during natural infection but are critical for achieving neutralization breadth.^23^

Current efforts to elicit CD4bs bNAbs have adopted a germline-targeting approach, which seeks to create immunogens that specifically bind and activate inferred germline (iGL) forms of CD4bs bNAbs isolated from infected individuals.^24^ This method has been explored for the VRC01 and IOMA subclasses within the V_H_1-2 gene-restricted class of CD4bs bNAbs, since they have defined and well-characterized features.^25^^,^^26^^,^^27^^,^^28^^,^^29^^,^^30^ Although VRC01-class bNAbs are more broad and potent, IOMA-class bNAbs are thought to have features that are more feasible to elicit (i.e., a more common CDRL3 length, lower levels of SHM, and a more easily achieved way to accommodate the N276_gp120_ glycan).^8^^,^^22^^,^^28^ However, similar to other CD4bs bNAb iGLs, IOMA iGL does not neutralize primary HIV-1 strains,^28^ underscoring the challenge of designing immunogens that guide affinity maturation toward broadly neutralizing activity. Recent studies reporting the design and testing of IOMA-targeting sequential immunization strategies described eliciting CD4bs epitope-specific responses in wild-type animals and demonstrated heterologous serum neutralization in knockin and wild-type mice.^28^ Furthermore, IOMA-like monoclonal antibodies isolated from these immunization studies developed mutations in CDRL1 that enabled the accommodation of the N276_gp120_ glycan.^28^ Thus, directing germline focus toward IOMA-class antibody precursors presents a possible vaccine tactic for eliciting CD4bs bNAbs.

To further inform IOMA-targeting immunogen design, we evaluated IOMA’s structural features and identified characteristics that contribute to IOMA’s neutralization potency and breadth. We engineered a library of IOMA variants with reversions of SHMs to their iGL residues to identify which SHMs play a role in IOMA’s neutralization function versus which do not. Using this information, we created IOMAmin variants that included the minimal SHMs necessary to achieve the same neutralization potency and breadth as IOMA. Analysis of a 3.9 Å single-particle cryoelectron microscopy (cryo-EM) structure of an IOMAmin variant bound to Env showed that, despite containing fewer SHMs, the structural interactions between the IOMAmin variant and Env resembled those observed in the previously characterized structure of mature IOMA bound to Env,^8^ validating our hypothesis that not all IOMA SHMs contribute to its ability to recognize and neutralize HIV-1. Additionally, we explored whether mutations to IOMA’s CDRH3 and CDRL1 could improve neutralization function. The results illuminate IOMA’s mechanism of neutralization and also inform the design of IOMA-targeting immunogens and the evaluation of potential IOMA-class bNAbs from natural infection or elicited by IOMA-targeting vaccine regimens.

## Results

### A fraction of SHMs contribute to IOMA’s neutralization potency and breadth

Although CD4bs bNAbs typically contain high levels of SHM,^13^^,^^14^^,^^18^^,^^21^^,^^22^ the design of minimally mutated CD4bs bNAbs and discovery of VRC01-class bNAbs with lower levels of SHM demonstrated that many SHMs are accessories of prolonged maturation during chronic infection and do not contribute to bNAb neutralization activity.^11^^,^^19^^,^^21^^,^^23^ Here, we sought to characterize the role of SHM in IOMA to understand how mutations influence neutralization and inform immunogen design efforts to elicit IOMA-class bNAbs. To identify SHMs in IOMA that contribute to its neutralization properties, we systematically designed a panel of IOMA variants in which individual SHMs were reverted to their iGL counterparts and evaluated the effects of these substitutions on neutralization against a pseudovirus screening panel.

**Figure 1:**
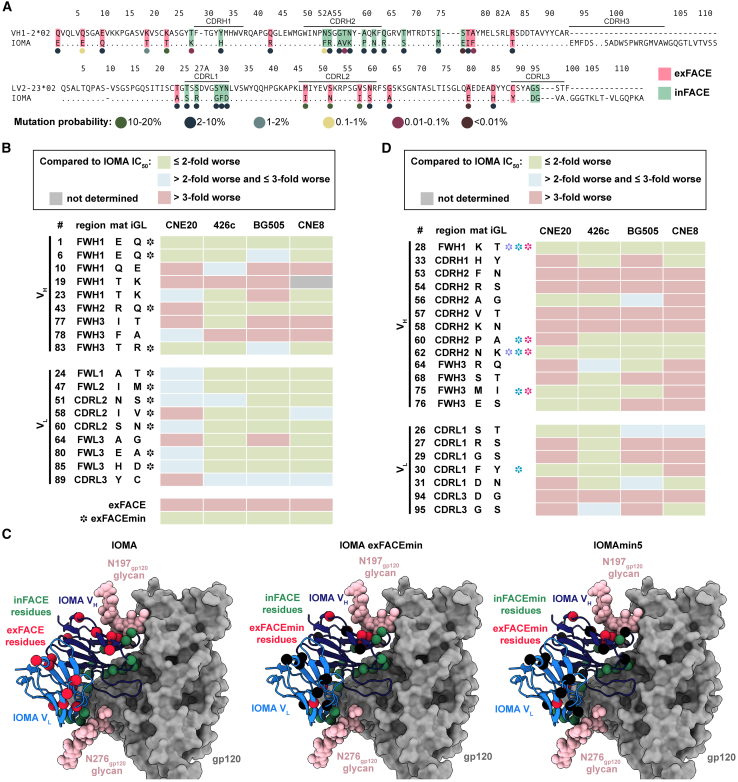
Screening SHMs that contribute to IOMA neutralization function (A) Sequence alignment of IOMA iGL and mature V_H_/V_L_ sequences with exFACE and inFACE SHM residues highlighted in red and green, respectively. Colored dots (colors defined in the legend below sequences) indicate mutation probabilities calculated by ARMADiLLO.^31^^,^^32^^,^^33^ Improbable mutations were defined as <1% probability.^31^^,^^32^^,^^33^ (B) Neutralization screening of IOMA exFACE variants against CNE20, 426c, BG505, and CNE8 strains. Black symbols () indicate exFACE mutations incorporated into the IOMAexFACEmin variant selected by the following criteria: IC_50_ values for each variant were ≤2-fold of IOMA’s IC_50_ against at least two strains and were >2-fold worse than mature IOMA against more than one strain. (C) Structural representations of IOMA, IOMAexFACEmin, and IOMAmin5 Fabs bound to gp120 with exFACE and inFACE residues shown as red and green spheres, respectively (PDB: 5T3X). Remaining SHMs are shown as black spheres. (D) Neutralization screening of IOMA inFACE variants against CNE20, 426c, BG505, and CNE8 strains. Purple symbols () indicate exFACE mutations incorporated into the IOMAmin3 variant selected by the following criteria: IC_50_ values for each variant were within 2-fold of IOMA against all four strains. Teal symbols () indicate exFACE mutations incorporated into the IOMAmin4 variant selected by the following criteria: IC_50_ values for each variant were within 2-fold of IOMA against at least three strains. Hot pink symbols () indicate exFACE mutations incorporated into the IOMAmin5 variant selected by the following criteria: IC_50_ values for each variant were within 2-fold of IOMA against at least three strains in the V_H_ and IC_50_ within 2-fold of IOMA against all four strains in the V_L_. IC_50_ values are represented as the average mean from duplicate neutralization measurements (*n* = 2). See also Figures S1 and S2.

We designated two cohorts of IOMA SHMs: SHMs that contribute to the antibody:Env interface and interact with Env gp120 residues, termed internal face SHMs (inFACE), and SHMs that do not contribute to antibody:Env interactions, namely external face SHMs (exFACE). The inFACE and exFACE residues were distinguished by analyzing the BG505-IOMA (PDB: 5T3X, 5T3Z) structures.^8^ SHM residues in the IOMA V_H_ and variable light (V_L_) domains containing an atom within 4.0 Å of a BG505 gp120 residue were considered inFACE residues, and the remaining SHMs were exFACE residues. This analysis assigned 9 V_H_/9 V_L_ exFACE residues and 13 V_H_/7 V_L_ inFACE residues (Figures 1A and S1A).

We first evaluated IOMA SHMs in the exFACE. Although these mutations were not involved in antibody:Env interactions, we hypothesized that some exFACE SHMs could contribute to maintaining IOMA’s structural integrity and thus indirectly contribute to neutralization potency and breadth. We created individual V_H_/V_L_ IOMA exFACE variants and tested neutralization against a small screening panel composed of pseudovirus strains that IOMA neutralizes with varying potencies (Figure 1B). Our panel included pseudoviruses for strains CNE20 (IOMA IC_50_: <0.1 μg/mL), 426c (IOMA IC_50_: 0.1–0.99 μg/mL), BG505 (IOMA IC_50_: 1.0–9.9 μg/mL), and CNE8 (IOMA IC_50_: 10–50 μg/mL),^8^^,^^34^^,^^35^ which represent a spectrum of HIV-1 neutralization sensitivities to IOMA, ranging from highly sensitive to more resistant, across different clades.

Within V_H_, 4 of 9 exFACE variants (Q10E_HC_, T19K_HC_, I77T_HC_, and F78A_HC_) resulted in >3-fold decreases in IC_50_ compared to IOMA against 3 strains (Figure 1B). In the context of all SHMs in the exFACE, these 4 residues are in closest proximity to the N197_gp120_ N-glycan, suggesting that these mutations may have evolved to stabilize glycan interactions (Figure S1B). In the V_L_, 7 of 9 exFACE variants resulted in comparable neutralization potencies compared to IOMA in the screening panel, suggesting that exFACE SHMs in the light chain (LC) play a lesser role in neutralization function compared to SHMs in the V_H_ exFACE (Figure 1B). Only 3 exFACE V_L_ variants (I58V_LC_, A64G_LC_, and Y89C_LC_) exhibited >3-fold decreases in IC_50_ compared to IOMA against at least 1 strain (Figure 1B).

Based on these results, we designed an IOMAexFACEmin variant that incorporates the minimal number of SHMs in the exFACE required to maintain IOMA’s neutralization activity (Figure S1A). We applied the following criteria to select individual exFACE mutations: IC_50_ values for each variant must be ≤2-fold of IOMA’s IC_50_ against at least two strains and not >2-fold worse in neutralization potency against IOMA for more than one strain (at least two green boxes and no more than one blue or red box) (Figures 1B and 1C). These mutations were then combined to create the IOMAexFACEmin variant. To validate our design of IOMAexFACEmin, we compared IOMA’s neutralization to IOMAexFACEmin and IOMAexFACE, a variant with all exFACE SHMs reverted to iGL residues, against the screening panel. We found that IOMAexFACEmin exhibited IC_50_ values within 2-fold of IOMA’s IC_50_ against all strains, whereas IOMAexFACE had IC_50_ values that were 3-fold greater than those of IOMA against all 4 strains (Figure 1B). These results are consistent with our hypothesis that a subset of somatically hypermutated exFACE residues contribute to IOMA’s neutralization activity without directly interacting with Env residues.

We applied the same methodology to IOMA inFACE residues (Figure 1D). Given that these residues interact with Env gp120, we expected that reversions of inFACE SHMs would adversely impact neutralization. Indeed, 8 of 12 V_H_ inFACE variants resulted in >3-fold increases in IC_50_ values compared to IOMA against at least 2 strains. 4 of these 8 variants (F53N_HC_, R54S_HC_, V57T_HC_, and K58N_HC_) demonstrated weak neutralization against all strains (Figure 1D). Furthermore, 6 of 9 V_H_ inFACE variants within CDRH2 showed greatly impaired neutralization against at least 2 strains, indicating the importance of SHMs in this region during maturation of IOMA (Figure 1D). In the V_L_, 4 of 7 inFACE variants (R27S_LC_, G29S_LC_, D94G_LC_, and G95S_LC_) resulted in >3-fold increases in IC_50_ compared to IOMA against at least 2 strains (Figure 1D). In one of these variants, G29_LC_ was reverted to the iGL Ser residue. A glycine in this position is hypothesized to facilitate CDRL1 flexibility necessary to accommodate the N276_gp120_ glycan.^8^ Our results support the role of this substitution in IOMA’s neutralization function.

### IOMAmin variants show comparable neutralization potency and breadth to IOMA

Using results from the exFACE and inFACE single-site variant neutralization screens (Figures 1B and 1D), we designed IOMAmin variants with the minimum numbers of SHMs required to maintain IOMA’s neutralization potency and breadth. Variants were constructed systematically to identify and retain only the SHMs that improved neutralization by IOMA while reverting others to their iGL counterparts. The IOMAmin design process began by first incorporating the SHMs from the exFACEmin variant that had minimal effects on neutralization. Subsequently, inFACE SHMs were added selectively based on their impact on neutralization, allowing incremental building of variants with progressively fewer mutations.

We designed three variants based on the following neutralization criteria: IOMAmin3 included inFACE mutations with IC_50_ values within 2-fold of IOMA against all four strains (four green boxes), IOMAmin4 included inFACE mutations with IC_50_ values within 2-fold of IOMA against at least three strains (at least three green boxes), and IOMAmin5 included inFACE mutations with IC_50_ values within 2-fold of IOMA against all four strains (four green boxes) in V_H_ and only exFACE mutations in V_L_ (Figures 1C and S1A).

The resulting IOMA variants contained a fraction of SHMs as IOMA, which has 22% somatically hypermutated amino acid substitutions in V_H_ and 14% in V_L_. For IOMAexFACEmin, there were 18% and 8% somatically mutated amino acid substitutions in V_H_ and V_L_, respectively (Figure S2A). The level of SHM substitutions for IOMAmin3, IOMAmin4, and IOMAmin5 was even further reduced to 16% V_H_/8% V_L_, 14% V_H_/7% V_L_, and 14% V_H_/8% V_L_, respectively (Figure S2A). These variants contain the fewest SHM substitutions in the IOMA class of CD4bs antibodies and similar levels of SHM substitutions to “minimal” VRC01-class antibodies such as minVRC01 and 12a21min^21^ (Figures S2A and S2B). We also investigated the effects of substitutions at these positions on non-specific binding using an *in vitro* polyreactivity assay for evaluating IgGs,^36^ finding that IOMA variants were not polyreactive as compared to highly polyreactive HIV-1 bNAbs such as 4E10 and 45-46m2^37^^,^^38^ (Figure S2C).

To further assess the likelihood of eliciting the SHMs observed in IOMAmin variants, we performed an analysis using the ARMADiLLo (Antibody Residue Mutations Assessed by Deep Learning) web tool.^31^^,^^32^^,^^33^ ARMADiLLo evaluates the probability of individual SHMs based on observed mutational patterns in human antibody repertoires, allowing assessment of the improbability of these mutations.^31^^,^^32^^,^^33^ We found that while IOMAmin variants contain fewer improbable SHMs (5 in V_H_ and 0 in V_L_) compared to mature IOMA (7 in V_H_ and 1 in V_L_), there remained several improbable SHMs, particularly within CDRH2 and FWH3 (Figures 1, S1A, and S2A). In comparison to the number of improbable mutations in minVRC01 (6 in V_H_ and 8 in V_L_) and other VRC01-class bNAbs, IOMAmin variants exhibited a lower frequency of improbable mutations, especially in the V_L_ (Figure S2A). These findings support the feasibility of eliciting IOMA-like bNAbs through vaccination, as the SHMs required for neutralization potency and breadth are largely within the range of naturally observed mutations.

**Figure 2:**
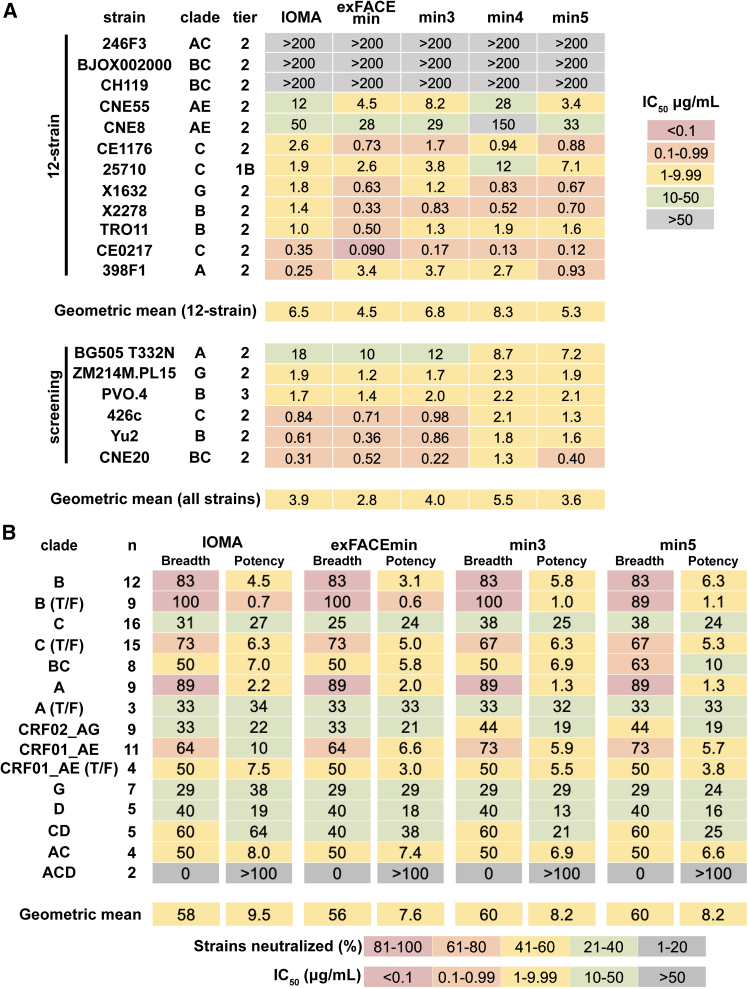
IOMAmin constructs show comparable neutralization profile to IOMA (A) Neutralization IC_50_ values for IOMA and IOMAmin variants against the global 12-strain viral panel^39^ and six additional screening strains. (B) Overview of neutralization for IOMA and IOMAmin variants against a cross-clade 119-strain panel grouped into clades, where n is the number of strains per clade. Breadth is indicated by the percentage of neutralized strains for each clade (IC_50_ ≤ 50 μg/mL), and potency is indicated by geometric mean IC_50_. IC_50_ values are represented as the average mean from duplicate neutralization measurements (*n* = 2).

We next evaluated IOMAexFACEmin, IOMAmin3, IOMAmin4, and IOMAmin5 against a global 12-strain HIV-1 pseudovirus neutralization panel^39^ and six additional screening strains (Figure 2A). Notably, IOMAexFACEmin and IOMAmin5 exhibited comparable or lower geometric IC_50_ mean values against a global 12-strain panel^39^ compared to IOMA (4.5 and 5.3 μg/mL versus 6.5 μg/mL), while IOMAmin3 and IOMAmin4 showed comparable or higher geometric mean values against the 12-strain panel to IOMA (6.8 and 8.3 μg/mL versus 6.5 μg/mL). These trends in geometric mean IC_50_ values were consistent across all 18 strains (Figure 2A). IOMAmin4 exhibited the weakest geometric mean IC_50_s in both comparisons, suggesting a potential role of the F30Y_LC_ SHM in neutralization. Overall, these findings highlight the retention of neutralization activity for IOMAmin variants despite harboring fewer SHMs.

We also evaluated IOMAexFACEmin, IOMAmin3, and IOMAmin5 against a cross-clade 119-strain pseudovirus panel to evaluate their potencies and breadth compared to mature IOMA, finding that all three variants showed improvements in potency compared to IOMA (Figure 2B). IOMAexFACEmin exhibited the highest potency with a geometric mean IC_50_ of 7.6 μg/mL, comparable to IOMA’s mean IC_50_ of 9.5 μg/mL. IOMAmin3 and IOMAmin5 also showed slight improvements in breadth, each neutralizing 60% of strains compared to IOMA’s 58%. These results emphasize the capacity of the IOMAmin variants to maintain, and even marginally enhance, their neutralization potencies and breadth despite having fewer SHMs.

### Structure of an IOMAmin5-Env complex shows a different CDRH3 architecture from IOMA

**Figure 3:**
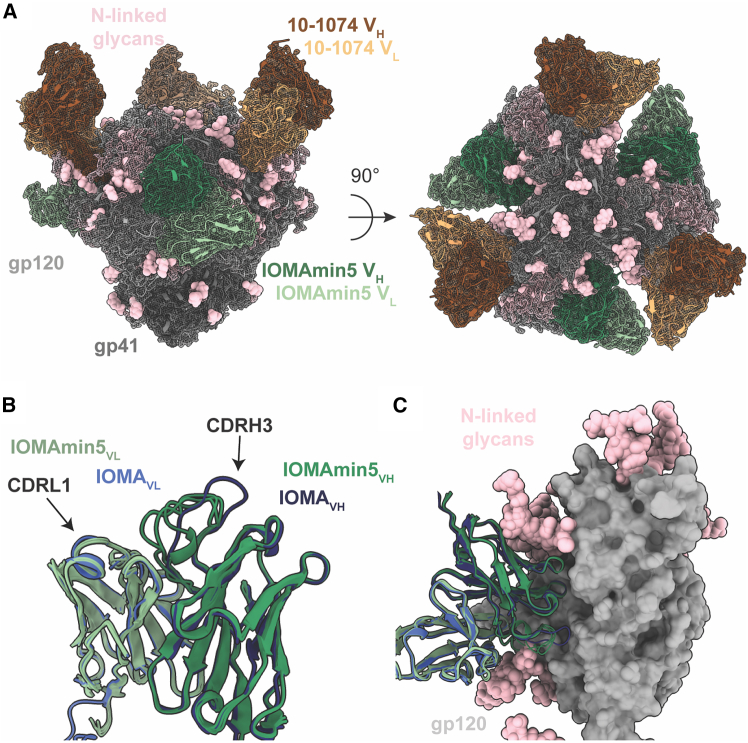
Single-particle cryo-EM structure of the IOMAmin5-Env complex reveals altered CDRH3 architecture (A) Side and top views of 3.9-Å single-particle cryo-EM density (mesh) and model (cartoon representation) of the IOMAmin5–BG505–10-1074 complex. N-linked glycans are represented as pink spheres. (B) Structural alignment of IOMAmin5 (this study) and mature IOMA (PDB: 5T3X) Fabs (cartoon representation) with black arrows pointing to CDRL1 and CDRH3 loops. (C) Structural alignment of IOMAmin5 and IOMA V_H_-V_L_ domains (cartoon representations) bound to gp120 (surface representation) (PDB: 5T3X). See also Figure S2 and Table S1.

To evaluate the recognition of HIV-1 Env by IOMAmin5, we solved two single-particle cryo-EM structures of IOMAmin5 and 10-1074 Fabs bound to BG505 SOSIP Env, facilitating comparisons with a previously described crystal structures of an IOMA and 10-1074 Fabs complexed with BG505^8^ (Figures 3A, S3A, and S3B). Class I (3.9 Å resolution) revealed density for three IOMAmin5 and three 10-1074 Fabs bound to BG505, while class II (4.2 Å) displayed density for only two IOMAmin5 and three 10-1074 Fabs bound to BG505 (Figures S3C–S3F). Both classes exhibited C1 symmetry. Notably, despite the approximate C3 symmetry in the class I structure, asymmetry was evident in the IOMAmin5 V_H_ CDRH3 as each of the three IOMAmin5 CDRH3 regions adopted distinct disordered loops (Figure 3B). By contrast, the mature version of IOMA’s CDRH3, although also disordered, extends toward the CD4bs,^8^ unlike the CDRH3 loops observed for IOMAmin5 (Figure 3C). These differences may stem from the distinct sequences of IOMAmin5 and IOMA or from intrinsic disorder of CDRH3. Potential variations in the CDRH3s of mature IOMA bound to BG505 SOSIP could not be addressed because available structures of IOMA-BG505 complexes were derived from X-ray crystallographic analyses of complexes in which 3-fold symmetry was imposed by crystal packing.^8^

A distinguishing characteristic of IOMA-class antibodies involves their mechanism for accommodating the N276_gp120_ N-glycan through CDRL1.^8^ In contrast to VRC01-class CD4bs bNAbs, which require a two- to six-residue deletion or selection of multiple glycines within CDRL1 to accommodate the N276_gp120_ glycan, IOMA-like bNAbs do not require such insertions or deletions.^8^^,^^13^^,^^14^^,^^18^ Instead, they rely on substitutions that enable a longer CDRL1 to provide the necessary flexibility to accommodate this glycan. The observation that IOMAmin5 adopts an identical CDRL1 conformation to that of mature IOMA (Figures 3B and 3C) suggests that sequence differences between IOMA and IOMAmin5 that are outside of CDRL1 (the CDRL1 sequences are identical) do not influence the CDRL1 conformation.

### IOMAmin5 exhibits CD4bs recognition comparable to that of IOMA

**Figure 4:**
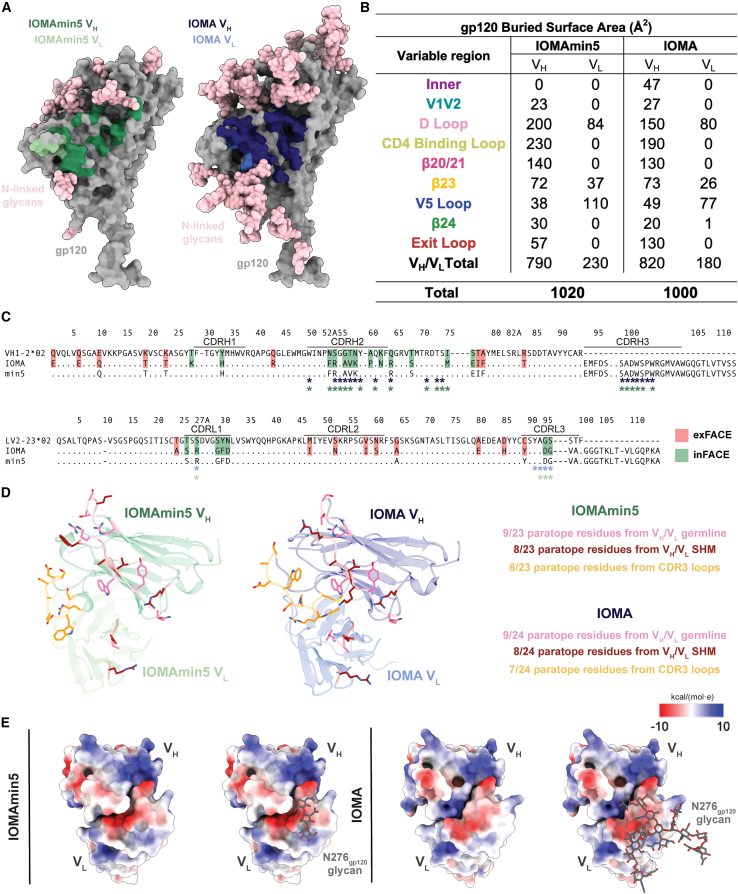
IOMAmin5 and mature IOMA exhibit similar gp120 CD4bs recognition (A) Surface contacts made by IOMAmin5 V_H_ and V_L_ on BG505 gp120 (left) and surface contacts made by IOMA V_H_ and V_L_ on BG505 gp120 (right). (B) Summary of gp120 buried surface area (BSA) (Å^2^) calculations for IOMAmin5 and IOMA on regions of gp120: inner domain (inner), D loop, CD4bs loop, β20/21, β23, V5 loop, β24, and exit loop of the CD4bs. BSA calculations were conducted for gp120 protein components and did not include glycan interactions. (C) Sequence alignment of IOMA and IOMAmin5 (min5) constructs with iGL sequences. exFACE and inFACE SHM residues are highlighted in red and green, respectively. IOMA and IOMAmin5 V_H_/V_L_ residues that contribute to the paratope are labeled with an asterisk below the sequence alignment in blue (IOMA) and green (IOMAmin). (D) IOMA and IOMAmin5 paratope residues within CDR3 loops and from germline V genes or an SHM highlighted as sticks. (E) Surface representations of IOMAmin5 (left) and IOMA (right) V_H_-V_L_ domains with electrostatic potentials (kcal/[mol·*e*]) colored blue (positive electrostatic potential) to red (negative electrostatic potential) shown without and with superimposed coordinates for the N276_gp120_ glycan shown as sticks. Values were calculated using ChimeraX Coulombic Surface Coloring.^40^

IOMAmin5 and IOMA buried comparable surface areas on BG505 gp120 (1,020 and 1,000 Å^2^, respectively); however, the distribution of buried surface area (BSA) on gp120 varied for IOMAmin5 and IOMA (Figures 4A and 4B). Unlike IOMA, IOMAmin5 showed no BSA in the gp120 inner domain, which includes the highly conserved K97_gp120_.^18^ Moreover, IOMA exhibited more than double the BSA in the gp120 exit loop compared to IOMAmin5, which had more concentrated BSA in the D and CD4 binding loops (Figure 4B). These distinctions in gp120 recognition may have contributed to slight variances in observed neutralization potencies (Figure 2).

A comparison of the paratopes of IOMAmin5 and IOMA revealed that residues engaged with the gp120 CD4bs were largely similar (Figure 4C). The most notable differences were observed in the CDRH2s, where IOMAmin5 exhibited interactions not seen in IOMA, utilizing F52_HC_, attributed to SHM, along with I75_HC_, which was one of the SHM reversions incorporated into the IOMAmin5 design. Both IOMAmin5 and IOMA utilized nearly 40% of the paratope surface for residues encoded by V_H_ and V_L_ germline genes and ∼30% for residues altered by SHM (Figure 4D). The remaining ∼30% of the paratope surfaces involved CDR3 loops (Figure 4D).

In addition to comparing the epitopes and paratopes of the IOMAmin5-BG505 and IOMA-BG505 structures, we evaluated potential electrostatic changes in the V_H_/V_L_-gp120 interfaces. Prior studies noted a shift toward a more positively charged antigen combining site during the evolution of CD4bs-targeting bNAbs.^41^ This alteration was postulated to facilitate the accommodation of negatively charged sialic acids on complex-type N-glycans in gp120.^41^ Although IOMAmin5 does not represent a naturally occurring precursor of IOMA, we were interested in determining if the reversals of SHMs led to variations in the electrostatic potential of the antibody paratope. We found that regions of V_L_ near the N276_gp120_ glycan exhibited a somewhat more negative electrostatic potential for IOMAmin5 than for IOMA, suggesting a potential adaptation in IOMA to better accommodate the N276_gp120_ glycan, a complex-type N-glycan in BG505 SOSIP that includes negatively charged sialic acids (Figure 4E).^42^

### Additional substitutions in CDRH3 and CDRL1 do not enhance neutralization by IOMA

**Figure 5:**
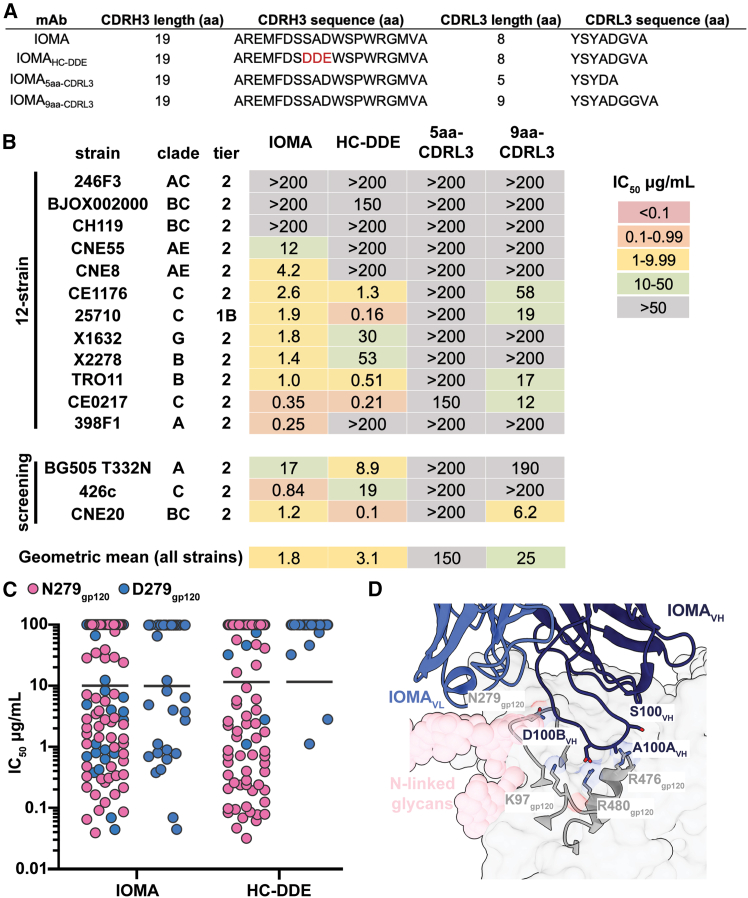
CDRH3 and CDRL1 mutations fail to improve IOMA neutralization (A) Sequence alignment of CDRL1s and CDRL3s for IOMA and IOMA CDR variants. (B) Neutralization data for IOMA and IOMA CDR variants against a global 12-strain panel^39^ and three additional screening strains. (C) Neutralization IC_50_ profile of IOMA and IOMA_HC-DDE_ against a cross-clade 119-strain pseudovirus panel. Each symbol represents a unique HIV-1 isolate. Pink symbols represent isolates with N279_gp120_, and blue symbols represent isolates with D279_gp120_. Geometric means are shown as solid black bars. IC_50_ values are represented as the average mean from duplicate neutralization measurements (*n* = 2). (D) Interactions of IOMA CDRH3 with BG505 gp120 interface (PDB: 5T3X).

In addition to optimizing IOMA’s level of SHM, we also engineered IOMA variants with modifications in CDRH3 and CDRL3 to investigate potential improvements in neutralization. The first variant, IOMA_HC-DDE_, included substitutions for CDRH3 residues S100_HC_, A100A_HC_, and D100B_HC_ to the negatively charged DDE motif (Figure 5A), a motif that was first described for ACS103, an IOMA-class bNAb that was isolated from a person infected with HIV-1 who was characterized as an elite neutralizer.^22^ In addition, the DDE and similar motifs were identified in monoclonal antibodies isolated from an IOMA germline-targeting vaccination study in IOMA iGL knockin mice.^28^ The latter study suggested that adoption of the DDE motif might have been driven by a well-conserved cluster of positively charged residues present at the IOMA-contacting interface of the Envs utilized during the immunization regimen (K97_gp120_ [90% conserved], R476_gp120_ [R: 64% conserved; R/K: 98% conserved], and R480_gp120_ [99% conserved]).^28^ To assess the relevance of CDRL3 features in IOMA antibodies, we designed two IOMA variants with modifications in CDRL3. One variant, IOMA_5aa-CDRL3_, featured a 5-residue CDRL3 akin to the VRC01 class of bNAbs known for its broad and potent neutralization (Figure 5A).^13^^,^^14^^,^^18^^,^^20^^,^^41^ The other, IOMA_9aa-CDRL3_, featured a 9-residue CDRL3, a length that is nearly 8 times more prevalent in the human B cell repertoire than an 8-residue CDRL3 (Figure 5A).

We evaluated neutralization potencies of these variants in a global 12-strain HIV-1 pseudovirus neutralization panel^39^ plus three additional screening strains (Figure 5B). IOMA_5aa-CDRL3_ lost neutralization capabilities against all strains, while IOMA_9aa-CDRL3_ suffered a 10-fold increase in geometric mean IC_50_ compared to IOMA (25 versus 1.8 μg/mL). IOMA_HC-DDE_ had a slightly increased geometric mean IC_50_ compared to IOMA (3.1 versus 1.8 μg/mL), showing varied performance against different strains. To further explore this finding, we tested IOMA_HC-DDE_ against the cross-clade 119-strain pseudovirus panel and found that IOMA_HC-DDE_ is N279_gp120_ dependent (Figure 5C). This observation was unexpected, given that N279_gp120_ is not proximal to the well-conserved cluster of positively charged residues in the CD4bs hypothesized to interact with the DDE motif (Figure 5D).^28^ This result could suggest that the DDE motif does not interact with the CD4bs in the hypothesized manner, possibly due to inherent flexibility in CDRH3, or that the DDE substitutions in this variant alter the conformation of CDRH3.

## Discussion

The identification and characterization of bNAbs against conserved HIV-1 Env epitopes provided a framework that led to engineering of immunogens to elicit bNAbs against particular epitopes and/or bNAbs of a specific class.^27^^,^^29^^,^^43^^,^^44^^,^^45^^,^^46^ Immunogen design to elicit bNAbs that bind the CD4bs is of particular interest since CD4bs bNAbs have been well characterized and are among the most broad and potent anti-HIV-1 bNAbs.^18^^,^^24^ However, the CD4bs epitope involves a recessed pocket that is framed by bulky N-linked glycans, presenting steric challenges that require CD4bs bNAbs to adopt rare features, further challenging vaccine design efforts.^18^^,^^23^^,^^24^^,^^44^^,^^45^^,^^47^

In this study, we focused on the IOMA class of CD4bs bNAbs, which represent a promising target due to their distinct characteristics: lower levels of SHM compared to most CD4bs bNAbs, an 8-amino acid CDRL3, and fewer mutations in CDRL1 to accommodate the N276_gp120_ glycan.^8^^,^^22^^,^^28^ We further characterized IOMA’s neutralization mechanism by evaluating the role of SHMs. We identified 5 of 9 V_H_ and 2 of 9 V_L_ exFACE SHMs that contribute to neutralization by IOMA but are not predicted to interact with Env gp120 residues. In the V_H_ domain, the proximity of these mutations to the N197_gp120_ glycan suggested that they evolved to stabilize and/or accommodate this glycan. Furthermore, we found that almost half of all V_L_ SHMs were in the exFACE and appeared to play no role in neutralization by IOMA. This suggested that V_L_ accumulated “passenger” mutations, likely resulting from IOMA’s prolonged SHM process. For inFACE SHMs that were predicted to contact gp120 residues, we found that most mutations in V_H_ and V_L_ contribute to IOMA’s neutralization activity. In particular, inFACE SHMs in CDRH2, which engage β24 and the CD4bs motifs in gp120, and in CDRL1, adjacent to the N276_gp120_ glycan when IOMA interacts with Env, proved to be necessary for IOMA’s potency and breadth, further suggesting that N-glycans in the gp120 CD4bs stimulated the evolution of IOMA’s SHMs.

With these results, we generated three IOMAmin variants that each included a fraction of the SHM substitutions found in mature IOMA but maintained its neutralization potency and breadth, suggesting that relatively few SHMs are needed for IOMA-class cross-neutralizing activity. We found that while IOMAmin variants included a similar number of SHMs as minVRC01^21^ (Figure S2A), the SHMs in IOMAmin variants are relatively more achievable as suggested by the ARMADiLLo analysis (Figure S2A).^31^^,^^32^^,^^33^ Furthermore, there were notable differences in the distribution of these substitutions (Figure S2B). IOMAmin variants include only essential SHMs, predominantly in CDRH2 and CDRL1, which are critical for accommodating key glycan interactions and maintaining neutralization activity (Figure S2B). By contrast, minVRC01 required mutations across CDRH1, CDRH2, and CDRL1, including deletions in CDRL1 to accommodate the N276_gp120_ glycan (Figure S2B).^21^ Additionally, minVRC01 depends heavily on its rare five-residue CDRL3,^13^^,^^14^^,^^20^ a feature absent in IOMAmin variants, which retain a more commonly observed eight-residue CDRL3.^8^^,^^18^ These distinctions highlight the divergent evolutionary pressures and germline precursor accessibility between the VRC01 and IOMA antibody classes.

These results are useful for identifying sequences of IOMA-like antibodies derived from human patients and animal studies because they can guide and possibly predict specific substitutions that are important for broad and potent neutralization. Furthermore, our findings offer concrete guidance for the design of germline-targeting immunogens and sequential immunization strategies that aim to elicit IOMA-class bNAbs. With respect to this point, we recently demonstrated that IOMA IGT2 and IGT1 immunogens effectively engaged IOMA iGL and elicited IOMA-like, heterologously neutralizing antibodies in transgenic IOMA knockin mice.^28^ Sequencing data from monoclonal antibodies isolated from these mice revealed that several of the SHMs identified as critical in our current analysis were selected, for e.g., K19T_HC_, key mutations in CDHR2, and CDRL1 mutations at residues 29-31_LC_.^28^ However, fewer substitutions were elicited in FWH3, including critical residues S76E_HC_, T77I_HC_, and A78F_HC_.^28^ Our analysis shows that reverting the SHM at residue 78_HC_, which changes the phenylalanine at this position back to the germline alanine residue, resulted in weaker neutralization potencies across the four screening strains (Figure 1B). Notably, three of four substitutions in closest proximity to the N197_gp120_ glycan (E10Q_HC_, T77I_HC_, and A78F_HC_), were largely absent from the elicited antibodies,^28^ suggesting incomplete SHM of these critical sites. Within CDRL1, while each elicited antibody included a single substitution within residues 29-31_LC_,^28^ our data highlight the importance of T26S_LC_, S27R_LC_, and substitutions of residues S29G_LC_, D30F_LC_, and N31D_LC_ for achieving optimal neutralization potency and breadth. These mutations likely support the accommodation of the N276_gp120_ glycan, as they are positioned in CDRL1, a region known to interact with this glycan in CD4bs bNAbs,^8^^,^^18^^,^^41^^,^^48^^,^^49^ and prior structural studies indicated that mutations in this region can facilitate steric compatibility with the glycan, promoting more favorable binding interactions with Env.^8^ Additionally, the critical exFACE SHM G64A_LC_ was absent from all of the elicited antibodies.^28^ From these observations, we suggest that future immunogen design strategies prioritize features that select for critical SHMs identified in our analysis, particularly (1) those stabilizing V_H_ domain residues near the N197_gp120_ glycan, (2) those accommodating the N276_gp120_ glycan through CDRL1 mutations, and (3) those in CDRH2 that engage β24 and CD4bs motifs in gp120, which may be important for enhancing IOMA-class neutralization breadth. Refining immunogens to guide these specific maturation pathways could enhance the elicitation of IOMA-class bNAbs with improved neutralization breadth and potency.

Our findings also underscore the limitations of engineering specific antibody features without considering their functional consequences. For example, attempts to incorporate a VRC01-like five-residue CDRL3 or a negatively charged CDRH3 DDE motif into IOMAmin variants failed to improve neutralization, emphasizing the importance of preserving lineage-specific structural adaptations (Figure 5B). The incorporation of the DDE motif in CDRH3, hypothesized to enhance neutralization by engaging a conserved cluster of positively charged residues on Env (Figure 5C),^28^ instead skewed neutralization toward N279_gp120_ dependency and thus reduced neutralization breadth. This observation is particularly noteworthy considering that only half of Env strains contain Asn at position 279_gp120_.^35^ Similarly, the selection of the DDE motif by the human antibody ACS103 during a naturally occurring HIV-1 infection demonstrated that these residues conferred an advantage in protecting from circulating HIV-1 strains in that individual.^22^ However, the decreased breadth of ACS103 compared to IOMA^22^ suggests that the presence of the DDE motif does not increase breadth or potency across diverse HIV-1 strains, consistent with results presented here. The implications of this outcome underscore the complexity of antibody-Env interactions and highlight the need for a more comprehensive understanding of factors influencing neutralization breadth and potency.

Together, the identification of mutations in IOMA that confer bNAb neutralization activity reveals viral pressures that influenced IOMA’s development. By identifying critical SHMs, we have taken an important step toward designing CD4bs-targeting immunogens capable of eliciting neutralizing antibodies with increased breadth and potency across diverse HIV-1 strains. Future studies can expand on this work by evaluating how these principles apply to other antibody lineages and integrating structural insights into iterative vaccine design strategies.

### Limitations of the study

A limitation of this study is that the analysis of SHMs is focused on regions outside of the CDRH3. This is because the germline sequence of IOMA was inferred, as the true germline precursor sequence is unknown, thus preventing a direct analysis of mutations within CDRH3. This limitation is notable because the CDRH3 region of IOMA plays a critical role in contacting Env and contributing to its neutralization mechanism. Future studies could focus on experimentally validating iGL sequences and exploring the specific contributions of CDRH3 residues to better understand how these mutations impact binding and neutralization breadth.

Additionally, our study evaluated the impact of SHMs using a panel of four HIV-1 strains (CNE20, 426C, BG505, and CNE8), which were selected to capture a range of neutralization sensitivities to IOMA and represent different HIV-1 clades. These strains were chosen as they provided a rational screening panel for systematically assessing the effects of individual SHM reversions on neutralization. The IOMAmin variants were designed based on performance across this panel and, notably, when tested against a larger 119-strain panel, IOMAmin variants retained neutralization breadth and potency, supporting the validity of this approach and the relevance of our initial screening strains in guiding minimal SHM selection.

Finally, while this study provides insights into how critical SHMs contribute to IOMA’s neutralization function, the efficacy of IOMA versus IOMAmin variants in protecting against viral challenge *in vivo* remains untested. The minimally mutated bNAbs minVRC01 and min12a21^21^ also have not been evaluated for protection efficacy from viral challenge. Such experiments would contribute to increased understanding of the relevance of minimally mutated bNAbs for vaccine design.

## Resource availability

### Lead contact

Correspondence and requests for additional information should be directed to Pamela J. Bjorkman (bjorkman@caltech.edu).

### Materials availability

Requests for plasmids for the IOMA antibodies reported in this paper for non-commercial research purposes should be directed to Pamela J. Bjorkman (bjorkman@caltech.edu).

### Data and code availability

Cryo-EM maps and atomic structures were deposited in the Protein DataBank (PDB) and Electron Microscopy DataBank under accession codes 9EHL and EMD-48059 for the structure of IOMAmin5–BG505–10-1074 class I and under accession codes 9EHM and EMD-48060 for the structure of IOMAmin5–BG505–10-1074 class II.

## Acknowledgments

We thank J. Vielmetter, A. Rorick, K. Storm, A. Lam, and the Protein Expression Center in the Beckman Institute at Caltech for assistance with expression and Z. Wu and J. Keeffe for help with polyreactivity assays. Electron microscopy was performed in the Caltech Cryo-EM Center with assistance from S. Chen. This work was supported by the 10.13039/100000060National Institute of Allergy and Infectious Diseases Grant HIVRAD P01 AI100148 (P.J.B.) and 10.13039/100000002NIH 1U54AI170856 (P.J.B.). The contents of this publication are solely the responsibility of the authors and do not necessarily represent the official views of NIAID or NIH. This work was also supported in part by the 10.13039/100000865Bill & Melinda Gates Foundation grants INV-002143 (P.J.B.) and INV-036842 (M.S.S.). Under the grant conditions of the Foundation, a Creative Commons Attribution 4.0 Generic License has already been assigned to the Author Accepted Manuscript version that might arise from this submission.

## Author contributions

Conceptualization, K.A.D., H.B.G., and P.J.B.; methodology, K.A.D., Y.E.L., Z.Y., P.N.P.G., and M.S.S.; formal analysis, K.A.D. and A.P.W., Jr.; investigation, K.A.D., Y.E.L., Z.Y., P.N.P.G., and M.S.S.; resources, M.S.S. and P.J.B.; data curation, K.A.D. and Z.Y.; writing – original draft, K.A.D. and P.J.B.; writing – review and editing, K.A.D., H.B.G., A.P.W., Jr., M.S.S., and P.J.B.; visualization, K.A.D.; supervision, K.A.D., H.B.G., and P.J.B.; project administration, K.A.D. and P.J.B.; funding acquisition, M.S.S. and P.J.B.

## Declaration of interests

The authors declare no competing interests.

## STAR★Methods

### Key resources table

**Table:** 

REAGENT or RESOURCE	SOURCE	IDENTIFIER
**Antibodies**		

IOMA IgG	N/A	Gristick et al.^8^
IOMA exFACE and inFACE single-site IgG variants	This paper	N/A
IOMA exFACEmin IgG	This paper	N/A
IOMAmin3 IgG	This paper	N/A
IOMAmin4 IgG	This paper	N/A
IOMAmin5 IgG	This paper	N/A
IOMA_HC-DDE_ IgG	This paper	N/A
IOMA_5aa-CDRL3_ IgG	This paper	N/A
IOMA_9aa-CDRL3_ IgG	This paper	N/A
Goat anti-human IgG (H + L), HRP-conjugated	GenScript	Cat# A00166

**Bacterial and virus strains**		

Baculovirus particles (used in polyreactivity assay ELISA)	Protein Expression Center, Caltech	See Hötzel et al.^36^
Screening panel: HIV-1 Env-pseudotyped viruses (CNE20, 426C, BG505, CNE8, ZM214M.PL15, PVO.4, Yu2)	NIH AIDS Reagent Program	N/A
Global panel: 12 HIV-1 Env-pseudotyped viruses	NIH AIDS Reagent Program	Cat# 12670
Cross-clade panel: 119 HIV-1 Env-pseudotyped viruses	Seaman Lab	N/A

**Chemicals, peptides, and recombinant proteins**		

Bovine Serum Albumin (BSA)	Sigma-Aldrich	Cat# A9647
SuperSignal™ ELISA Femto Substrate	Thermo Fisher	Cat# 37074

**Deposited data**		

IOMAmin5-BG505-10-1074 class I - Cryo-EM map and atomic coordinates	This paper	PDB: 9EHL; EMDB EMD-48059
IOMAmin5-BG505-10-1074 class II - Cryo-EM map and atomic coordinates	This paper	PDB: 9EHM; EMDB EMD-48060

**Experimental models: Cell lines**		

Expi293 Expression System	Thermo Fisher	Cat# A14527
TZM.bl cells	NIH AIDS Reagent Program	Cat# 8129

**Recombinant DNA**		

Plasmids encoding IOMA, IOMAmin, and other IOMA variants	This paper	N/A
Plasmids encoding BG505 SOSIP.664	Sanders et al.^50^	N/A

**Software and algorithms**		

PyMOL	Schrödinger, LLC	https://pymol.org
UCSF ChimeraX	UCSF	https://www.cgl.ucsf.edu/chimerax/
Phenix	PHENIX Consortium; Adams et al.^51^ and Afonine et al.^52^	https://phenix-online.org
Coot	Emsley et al.^53^	https://www2.mrc-lmb.cam.ac.uk/personal/pemsley/coot/
RELION 3	Zivanov et al.^54^	https://www2.mrc-lmb.cam.ac.uk/relion/index.php/Main_Page
CryoSPARC	Structura Biotechnology; Punjani et al.^55^	https://cryosparc.com
MotionCor2	Zheng et al.^56^	https://msg.ucsf.edu/motioncor2
Gctf	Zhang^57^	https://www.mrc-lmb.cam.ac.uk/kzhang/Gctf/
PDBePISA	EMBL-EBI; Krissinel and Henrick^58^	https://www.ebi.ac.uk/pdbe/pisa/
ARMADiLLo	Duke Human Vaccine Institute; Beem et al.^33^	https://armadillo.dhvi.duke.edu
HIV Antibody Database	Apple App Store, West et al.^35^	https://apps.apple.com/us/app/hiv-antibody-database/id1232472905

**Other**		

QuikChange II XL Site-Directed Mutagenesis Kit	Agilent	Cat# 200521
NEBuilder® HiFi DNA Assembly Master Mix	NEB	Cat# E2621
HiTrap MabSelect Protein A column	Cytiva	Cat# 17549851
HisTrap HP column	Cytiva	Cat# 29051021
Superdex 200 Increase 10/300 column	Cytiva	Cat# 28990944
Superose 6 Increase 10/300 GL column	Cytiva	Cat# 29091596
Nunc MaxiSorp 384-well plates	Thermo Fisher	Cat# 464718
Quantifoil R2/2 400 mesh Gold Grids	Ted Pella	Cat# 657-400-AU

### Experimental model and subject details

#### Cell lines

Expi293T cells (Thermo Fisher, Cat# A14527) were used for recombinant protein expression and maintained at 37°C with 8% CO_2_ in Expi293 Expression Medium (Thermo Fisher). Cells were cultured under constant shaking at 130 rpm and transfected using the Expi293 Expression System Kit according to the manufacturer’s protocol.

TZM-bl cells (obtained through the NIH AIDS Reagent Program, Cat# 8129) were used for pseudovirus neutralization assays. Cells were cultured at 37°C with 5% CO_2_ in Dulbecco’s Modified Eagle Medium (DMEM; Thermo Fisher) supplemented with 10% fetal bovine serum (FBS), 1 mM sodium pyruvate, 2 mM L-glutamine, and 1× antibiotic-antimycotic (Thermo Fisher).

All cell lines were of female origin, were not specifically authenticated, and routinely tested for mycoplasma contamination.

### Method details

#### Design of IOMA variants

Sites for IOMAmin exFACE and inFACE mutations were determined by analyzing the interactions between BG505 and IOMA in the BG505-IOMA-10-1074 structures (PDB: 5T3Z, 5T3X) in PyMol (Schrödinger LLC). IOMA residues that include atoms that were ≤4.0 Å from a BG505 residue were determined as inFACE residues and the remaining SHMs were defined as exFACE residues. Genes encoding IOMA exFACE and inFACE single-site variants and IOMA CDRL3 and DDE variants were generated using site-directed mutagenesis (Agilent QuikChange II XL Site-Directed Mutagenesis Kit, Cat#200521) starting with mature IOMA HC and LC genes, and IOMAexFACEmin and IOMAmin variants were generated using Gibson cloning (NEB NEBuilder HiFi DNA Assembly Master Mix, Cat# E2621).

#### Mutation probability analyses using ARMADiLLo

To assess the likelihood of SHMs in IOMAmin variants and other CD4bs bNAbs, we used ARMADiLLo (Antibody Residue Mutations Assessed by Deep Learning) (https://armadillo.dhvi.duke.edu/).^31^^,^^32^^,^^33^ This tool estimates the probability of observed mutations by analyzing large-scale human antibody repertoire datasets, allowing for classification of mutations as highly probable, moderately probable, or improbable. For our analyses, we input the heavy and light chain sequences of IOMA, IOMAmin variants (IOMAmin3, IOMAmin4, IOMAmin5), and other CD4bs bNAbs to compare the frequency of probable and improbable mutations across these antibodies. Each individual SHM was assessed, and the probability scores were extracted and categorized based on the tool’s built-in ranking system.

#### Protein expression and purification

Expression vectors encoding IgGs and Fabs were transfected using the transient Expi293 expression system (Thermo Fisher, Cat# A14527), according to the manufacturer’s protocol.^7^^,^^59^ Expression vectors included IgG HC or Fab HC and LC genes. Fab HC expression vectors encoded a C-terminal 6x-His tag. Expressed proteins were isolated from cell supernatants from IgG and Fab transfections using HiTrap MabSelect Protein A (Cytiva, Cat# 17549851) and Ni^2+^-NTA (Cytiva, Cat# 29051021) affinity chromatography for IgGs and Fabs, respectively. Subsequently, IgG and Fabs were purified over size exclusion chromatography (SEC) using a Superdex 200 Increase 10/300 column (Cytiva, Cat# 28990944). Proper folding of variants was assessed by SEC profiles and expression yields.

BG505 SOSIP.664 Env constructs encoded SOSIP mutations including disulfide mutations 501C and 605C (SOS), I559P (IP), and the furin cleavage site mutated to six arginine residues (6R).^50^ BG505 SOSIP.664 Env expression vectors were transfected using the transient Expi293 expression system (Thermo Fisher, Cat# A14527), according to the manufacturer’s protocol. Trimeric Env was separated from cell supernatants using PGT145 immunoaffinity chromatography and SEC using a Superose 6 Increase 10/300 GL column (Cytiva, Cat# 29091596).^50^^,^^60^

#### HIV-1 TZM.bl neutralization assays

Neutralization activities of IOMA-based IgGs were determined using a luciferase-based TZM.bl pseudovirus assay conducted using standard protocols.^39^^,^^61^ IC_50_ values were determined from independent replicates (*n* = 2) analyzed using Antibody Database (v2.0)^35^ with 5-parameter curve fitting. Non-specific activity was determined by evaluating IgGs against murine leukemia virus (MuLV).

#### Polyreactivity assay

A baculovirus-based polyreactivity assay was performed using an established enzyme-linked immunosorbent assay (ELISA) method to detect nonspecific binding.^36^ Briefly, a solution containing 1% baculovirus particles (Protein Expression Center, Caltech) in 100 mM sodium bicarbonate buffer (pH 9.6) was applied to the wells of a 384-well ELISA plate (Nunc MaxiSorp 384-well plates, Thermo Fisher, Cat# 464718) using a Tecan Freedom Evo liquid handling robot. Following overnight at 4°C incubation, plates were blocked for 1-h at room temperature with phosphate-buffered saline (PBS) containing 0.5% bovine serum albumin (BSA) (Sigma-Aldrich, Cat# A9647). Purified IgGs, diluted to 1 μg/mL in PBS with 0.5% BSA, were added to the blocked assay plate and incubated for 3 h at room temperature. Detection of bound IgG utilized a horseradish peroxidase–conjugated anti-human IgG (H + L) secondary antibody (GenScript, Cat# A00166), with luminescence signals measured at 425 nm with SuperSignal ELISA Femto Maximum Sensitivity Substrate (Thermo Fisher, Cat# 37074). Mean RLUs were determined from independent quadruplicates (*n* = 4).

#### Assembly of protein complexes and cryo-EM sample preparation

Protein complexes for cryo-EM were generated by combining purified IOMAmin5 and 10–1074 Fabs with a BG505 SOSIP.664 Env trimer in a 3:1 Fab:trimer molar ratio and incubating at 4°C overnight. Fab-Env complexes (3 μL) were applied to Quantifoil R2/2 400 mesh gold cryo-EM grids (Ted Pella, Cat# 657-400-AU) that were prepared by glow-discharging for 1 min at 20 mA using a PELCO easiGLOW (Ted Pella). Grids were blotted with Whatman No. 1 filter paper for 3 s at 100% humidity at room temperature and vitrified via plunge-freezing in liquid ethane using a Mark IV Vitrobot (Thermo Fisher).

#### Cryo-EM data collection

For the single-particle cryo-EM study of the IOMAmin5-BG505-10-1074 complex, data collection was performed using a Titan Krios transmission electron microscope operated at 300 kV. Movies were recorded with beam-image shift over a single exposure per hole in a 3-by-3 pattern of 2 μm holes. Movies were captured in super-resolution mode on a K3 camera (Gatan) equipped with a BioQuantum energy filter (Gatan) using a 20 eV slit width, yielding a pixel size of 0.4327 Å⋅pixel^−1^. The defocus range was set between 1.0 and 3.0 μm.

Data processing was performed with RELION software. Movies were motion corrected using MotionCor2^56^ after binning, and GCTF^57^ was used for contrast transfer function (CTF) estimation. Micrographs with poor CTF fits or ice quality were excluded. Manual particle picking was conducted for a subset of particles, followed by reference-free 2D classification. Selected 2D class averages were used for automated particle picking using the RELION AutoPicking module.^54^^,^^62^ Resulting particles were subjected to several rounds of 2D and 3D classifications. An initial model was generated using cryoSPARC^55^ with a subset of particles and used as a reference for 3D classification assuming C1 symmetry.

Two distinct classes were identified from 3D classification representing the IOMAmin5-BG505-10-1074 complex: class I exhibited density corresponding to three IOMAmin5 and three 10–1074 Fabs bound to BG505; class II showed density for two IOMAmin5 and three 10–1074 Fabs bound to BG505. These classes were subsequently refined using 3D auto-refinement and underwent post-processing in RELION.^54^^,^^62^ Particle polishing, CTF refinement, and multiple iterations of 3D auto-refinement were conducted to enhance map quality. Final high-resolution maps were generated and resolutions were determined in RELION^54^^,^^62^ based on the gold-standard Fourier shell correlation (FSC) criterion at 0.143. FSCs were computed using the 3DFSC program.^63^

#### Cryo-EM model building and refinement

Models were generated by fitting the coordinates of gp120 (PDB: 5T3X), gp41 (PDB: 5T3X), and 10–1074 Fab (PDB: 5T3X) into cryo-EM density maps using UCSF ChimeraX.^40^ Initial models were refined using the Phenix program’s real-space refinement module.^51^^,^^52^ Subsequent updates to the sequence and additional manual adjustments were carried out using Coot software.^53^ The refinement process involved iterative cycles of automated refinement using Phenix^51^^,^^52^ and manual adjustments in Coot to produce the final models (Table S1).^53^

#### Structural analyses

PyMol (Schrödinger LLC) and UCSF ChimeraX^40^ were used to prepare structure figures. PDBePISA^58^ was used to calculate BSAs using a 1.4 Ǻ probe. BSA calculations for gp120 were for its protein components and did not include contributions from glycans. Defined interactions were assigned tentatively due to the relatively low resolution of complexes using the following criteria: hydrogen bonds were assigned for pairwise interactions <4.0 Å and with an A-D-H angle >90° and van der Waals interactions were assigned as distances of <4.0 Å between atoms. Electrostatic potentials were calculated using the Coulombic Surface Coloring module in UCSF ChimeraX.^40^

### Quantification and statistical analysis

Neutralization assays were performed using a luciferase-based TZM.bl pseudovirus assay, with IC_50_ values determined from two independent replicates (*n* = 2) per variant-virus pair. Data analysis was conducted using the Antibody Database (v2.0),^35^ which applies a 5-parameter logistic curve fitting model. For all neutralization assays, the center is defined as the geometric mean, and dispersion is represented as fold-changes in IC_50_ values relative to mature IOMA. Statistical comparisons between antibody variants were based on relative differences in geometric mean IC_50_ values and breadth across different panels HIV-1 strains, as reported in the main text and figure legends (Figures 1B, 1D, 2A, 2B, 5B, and 5C).

The ARMADiLLo tool was used to assess the probability of each somatic hypermutation (SHM) in IOMA and IOMAmin variants, as described in the Methods section.^31^^,^^32^^,^^33^ Mutations were categorized as highly probable, moderately probable, or improbable based on the default thresholds established by ARMADiLLo, and results are summarized in Figures 1A, S1A, and S2A.^31^^,^^32^^,^^33^ The polyreactivity ELISA data (Figure S2B) were conducted in quadruplicates (*n* = 4) and are reported as mean relative luminescence units (RLU) ± standard error of the mean (SEM).

For cryo-EM data, map resolutions were determined using the gold-standard Fourier shell correlation (FSC) 0.143 criterion in RELION,^54^^,^^62^ and local resolution estimation was performed using 3DFSC.^63^ Resolution values and refinement statistics are reported in Table S1. Surface area calculations, electrostatic potential mappings, and structural alignments were performed using UCSF ChimeraX^40^ and PDBePISA.^58^ Electrostatic surfaces were visualized using the Coulombic Surface Coloring tool, and contact interfaces were defined by buried surface area (BSA) with a 1.4 Å probe radius.^40^^,^^58^

## Supplemental information

Document S1. Figures S1–S3 and Table S1

Document S2. Article plus supplemental information
