# Supplementary material for: Mapping essential somatic hypermutations in a CD4-binding site bNAb informs HIV-1 vaccine design
Source: Cell Rep. 2025 May 15;44(5):115713. doi: 10.1016/j.celrep.2025.115713 (PMC12117015; doi:10.1016/j.celrep.2025.115713)
Supplement: Document S1. Figures S1–S3 and Table S1 [file mmc1.pdf]

Cell Reports, Volume *44*

Supplemental information

Mapping essential somatic hypermutations in a CD4-binding site bNAb informs HIV-1 vaccine design

Kim-Marie A. Dam, Harry B. Gristick, Yancheng E. Li, Zhi Yang, Priyanthi N.P. Gnanapragasam, Anthony P. West Jr., Michael S. Seaman, and Pamela J. Bjorkman

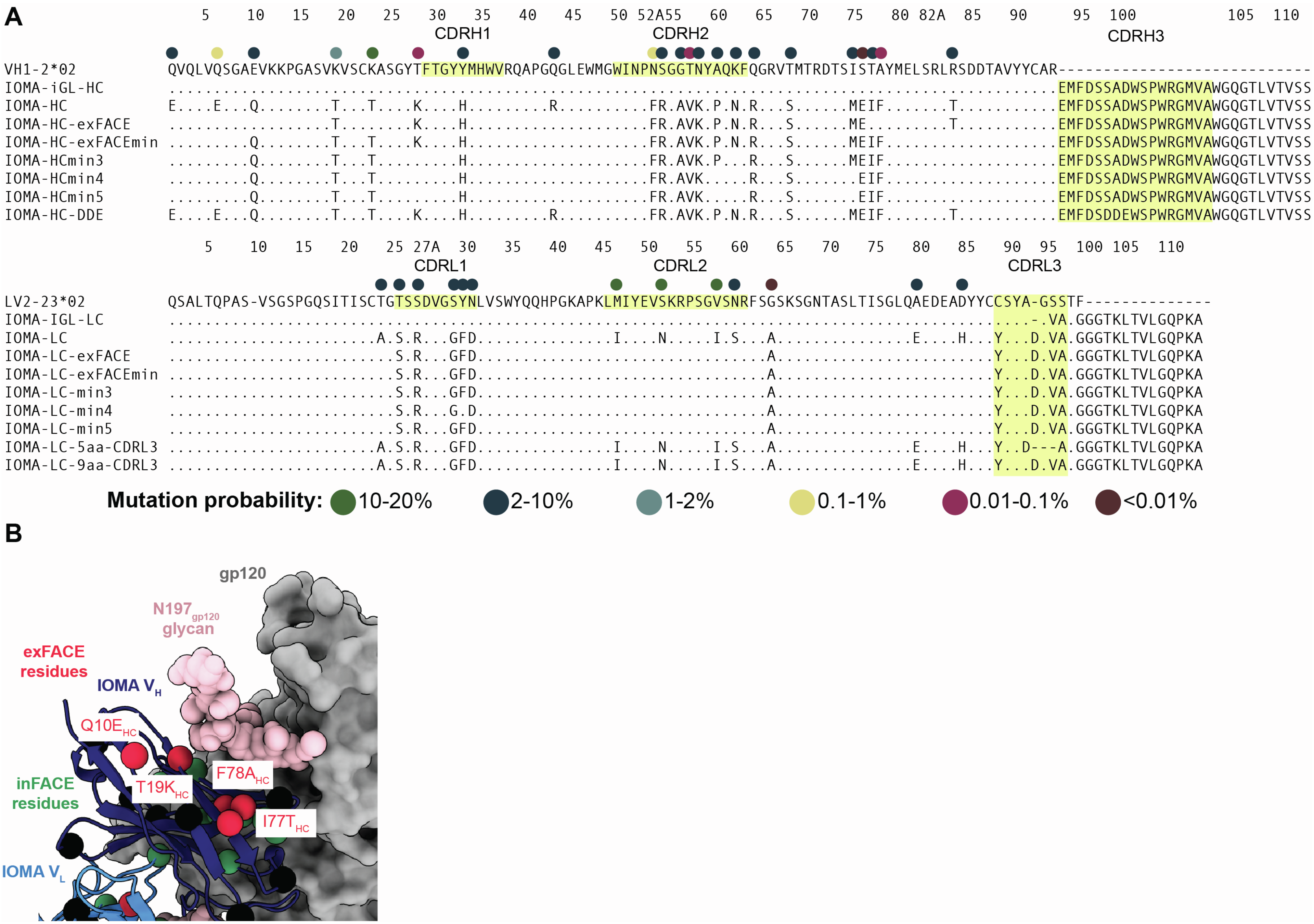
A
5 10 15 20 25 30 35 40 45 50 52A55 60 65 70 75 80 82A 85 90 95 100 105 110
CDRH1
CDRH2
CDRH3
VH1-2*02 QVQLVQSGAEVKKPGASVKVSCKASGYTFTGYYMHWVRQAPGQGLEWMGWINPNSGGTNYAQKFQGRVTMTRDTSISTAYMELSRLRSDDTAVYYCAR---------------------------
IOMA-iGL-HC ....EMFDSSADWSPWRGMVAWGQGTLVTVSS
IOMA-HC E....E..Q.......T..T...K...H.........R..........FR.AVK.P.N.R..S.....MEIF.......T...........EMFDSSADWSPWRGMVAWGQGTLVTVSS
IOMA-HC-exFACE .................T..T...K...H....................FR.AVK.P.N.R..S.....ME.........T...........EMFDSSADWSPWRGMVAWGQGTLVTVSS
IOMA-HC-exFACEmin .........Q.......T..T...K...H....................FR.AVK.P.N.R..S.....MEIF......................EMFDSSADWSPWRGMVAWGQGTLVTVSS
IOMA-HCmin3 .........Q.......T..T.......H....................FR.AVK.P...R..S.....MEIF......................EMFDSSADWSPWRGMVAWGQGTLVTVSS
IOMA-HCmin4 .........Q.......T..T.......H....................FR.AVK.....R..S......EIF......................EMFDSSADWSPWRGMVAWGQGTLVTVSS
IOMA-HCmin5 .........Q.......T..T.......H....................FR.AVK.....R..S......EIF......................EMFDSSADWSPWRGMVAWGQGTLVTVSS
IOMA-HC-DDE E....E..Q.......T..T...K...H.........R..........FR.AVK.P.N.R..S.....MEIF.......T...........EMFDSDDEWSPWRGMVAWGQGTLVTVSS
5 10 15 20 25 27A 30 35 40 45 50 55 60 65 70 75 80 85 90 95 100 105 110
CDRL1
CDRL2
CDRL3
LV2-23*02 QSALTQPAS-VSGSPGQSITISCTGTSSDVGSYNLVSWYQQHPGKAPKLMIYEVSKRPSGVSNRFSGSKSGNTASLTISGLQAEDEADYYCCSYA-GSSTF-------------
IOMA-IGL-LC ....-.VA.GGGTKLTVLGQPKA
IOMA-LC ......................A.S.R...GFD..............I....N.....I.S...A..............E....H...Y...D.VA.GGGTKLTVLGQPKA
IOMA-LC-exFACE ........................S.R...GFD..............................A..........................Y...D.VA.GGGTKLTVLGQPKA
IOMA-LC-exFACEmin ........................S.R...GFD..............................A..........................Y...D.VA.GGGTKLTVLGQPKA
IOMA-LC-min3 ........................S.R...GFD..............................A..........................Y...D.VA.GGGTKLTVLGQPKA
IOMA-LC-min4 ........................S.R...G.D..............................A..........................Y...D.VA.GGGTKLTVLGQPKA
IOMA-LC-min5 ........................S.R...GFD..............................A..........................Y...D.VA.GGGTKLTVLGQPKA
IOMA-LC-5aa-CDRL3 ......................A.S.R...GFD..............I....N.....I.S...A..............E....H...Y...D---A.GGGTKLTVLGQPKA
IOMA-LC-9aa-CDRL3 ......................A.S.R...GFD..............I....N.....I.S...A..............E....H...Y...D.VA.GGGTKLTVLGQPKA
Mutation probability: 10-20% 2-10% 1-2% 0.1-1% 0.01-0.1% <0.01%
B
gp120
N197gp120 glycan
exFACE residues
IOMA VH
Q10EHC
T19KHC
F78AHC
I77THC
inFACE residues
IOMA VL

Figure S1. Sequence alignment and characterization of IOMAmin antibodies, related to Figure 1.

(A) Sequence alignment of IOMA iGL, mature, and mutant VH/VL sequences with CDR regions (as defined by Kabat numbering) highlighted in yellow. Colored dots indicate mutation probability calculated by ARMADiLLO. [S1-3] Improbable mutations are defined as <1% probability. [S1-3] (B) Structural representation of IOMAexFACEmin Fab bound to gp120 with exFACE and inFACE residues shown as red and green spheres, respectively (PDB 5T3X). Remaining SHMs are shown as black spheres. exFACE mutations in closest proximity to the $N197_{gp120}$ glycan are labeled.

A

		# of SHM Subsitutions		Percent mutation		Improbable mutations	
		V_H	V_L	V_H %	V_L %	V_H	V_L
IOMA-class	IOMA	22	15	22	14	7	1
	IOMA exFACEmin	18	8	18	8	5	0
	IOMAmin3	16	8	16	8	5	0
	IOMAmin4	14	7	14	7	5	0
	IOMAmin5	14	8	14	8	5	0
	ACS101	27	18	27	19	9	6
	ACS102	35	16	35	17	N/A	N/A
	ACS103	24	20	24	21	N/A	N/A
VRC01-class	VRC01	41	23	41	25	11	9
	minVRC01	14	7	14	8	6	8
	12a21	31	20	31	22	14	5
	12a21min	17	8	17	9	10	3
	BG24	23	19	23	20	9	5
	PCIN63-71I1a	21	17	21	18	10	6
	N6	41	30	41	32	12	6
	3BNC117	34	23	34	24	9	6
	NIH45-46	44	27	39	25	12	9

C

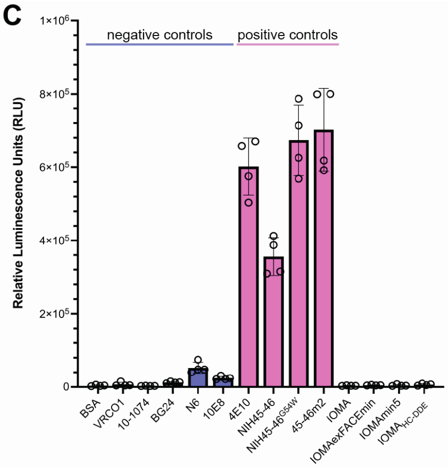

B

Variable Heavy

```
                                    CDRH1                  CDRH2                                                     CDRH3
VH1-2*02     QVQLVQSGAEVKKPGASVKVSCKASGYTFTGYYMHWVRQAPGQGLEWMGWINPNSGGTNYAQKFQGRVTMTRDTSI----STAYMELSRLRSDDTAVYYCAR----------------------------
IOMA         E....E...Q........T...T....K....H.........R..........FR.AVK.P.N.R...S.......M----EIF.......T...........EMFDSSADWSPWRGMVAWGQGTLVTVSS
IOMAmin5     .........Q........T...T.........H....................FR..VK.....R...S.......----EIF....................EMFDSSADWSPWRGMVAWGQGTLVTVSS
ACS101       E...LE............R...E.......K.FI........H....I....TLTS.V...RN....L.L...L.T----E.V..D.RN.K...........GGRGYDEPWGAYTWLDPWGQGSLVTVSS
ACS102       E.......GGLR......R...ET......K.FI...........K.I.L..TYTSAVKFERI......L...L.T----D.VH.D.KN.......I.....GGGGYDEPYY---TFDPWGQGSLVTVSS
ACS103       ............R.....R...E....R..A.HI..L..............V.TLTS.V..GW.......K...ER..DT--D...LD..G..L..........GNGKSDDERSTYQYFGPWGQGTLVTVSS
VRC01        ........GQM....E.MRI..R....E.IDCTLN.I.L...KRP.....LK.RG.AV...RPL.........VYS----D..FL..RS.TV.......F.T.GKNCD----YNWD-FEHWGRGTPVIVSS
minVRC01     ..............................CTLN................K.RF.AV...R...........VYS----D.....................GKNCD----YNWD-FEHWGRGTPVIVSS
NIH45-46     ..R.S...GQM....E.MRL..R....E.LNCPIN.I.L...RRP.....LK.RG.AV...R...........VYS----D..FL..RS.T.......F.T.GKYCTARDYYNWD-FEHWGRGAPVTVSS
3BNC117      ....L....A.T......R...E....NIRD.FI..W.........Q.V.....KT.QP.NPRQ.....SL..HA.WDFDTYSF..D.KA.........F...QRSDY---W----DFDVWGSGTQVTVSS
12a21        SQH.....TQ........R...Q.......N.IL..W.............L.K.VF.AV...RQ....IQL...IYR----EI.FLD..G............DESGDDLKWH----LHPWGQGTQVIVSP
min12a21     ..................R...........N.IL..W..............K.VF.AV...RQ..........IYR----EI.....................DESGDDLKWH----LHPWGQGTQVIVSP
N6           RAH.....TAM.......R...QT......AHILF.F.....R....V...K.QY.AV.FGGG.RD...L...VYR----EI...DIRG.KP..........DRSYGDSSWA----LDAWGQGTTVVVSA
PCIN63-71I1  ..................R..........NSCLI..W........Q..A....LH.AV...HQ....I.V.......----D......RG.......T...T.DASRDDRAWR----LDPWGQGTLVTVSS
BG24         .......R...............E....N.VDH.I.........RPQ.V..M..RG..V..S.R.................----D....Q.N..T.G........TQVKLDSSAGYP---FDIWGQGTMVTVSS
```

Variable Light

```
                                   CDRL1                  CDRL2                                   CDRL3
VL2-23*02    QSALTQPASVSGSPGQSITISCTGTSSDVGSYNLVSWYQQHPGKAPKLMIYEVSKRPSGVSNRFSGSKSGNTASLTISGLQAEDEADYYC-----------------
IOMA         ......................A.S.R...GFD...............I....N.....I.S...A...............E....H...YSYADGVAFGGGTKLTVL
IOMAmin5     ........................S.R...G...................................A.........................YSYADGVAFGGGTKLTVL
ACS101       SYE.....................S.--I...D.......Y.....VI.F..........H...........A.......V....I.H.YSYDDMIIFGGGTRLTVL
ACS102       ........A.................--I.GDH.......P..R....L....T.......H...........A.......L....T.H.CSFADMVIFGGGTSLTVL
ACS103       .TVV..................A.S...I...DY.............VV..D.T....DI.H...A...............P....Q.R.CTYAQTVLCGGGTKLTVL

VK3-20*01    EIVLTQSPGTLSLSPGERATLSCRASQSVSSSYLAWYQQKPGQAPRLLIYGASSRATGIPDRFSGSGSGTDFTLTISRLEPEDFAVYYC--------------
VRC01        .................T.II...T..Y---GS......R.......V..SG.T..A.........RW.P.YN....N..SG..G....QQYEFFGQGTKVQVD
minVRC01     ........A..............T..Y---GS................DG.N......A.....RW.P.......S...........QQYEFFGQGTKVQVD
NIH45-46     ........A........T.II...T...---GS......R.......V..SG.T..A.........RW.A.YN.S..N..SG..G....QQYEFFGQGTKVQVD
3BNC117      D.QM....SS..A.V.DTV.IT.Q.N-----G..N....RR.K..K....DG.KLER.V.S....RRW.QEYN...NN.Q...I.T.F.QVYEFVVPGTRL--D

VK1-33*01    DIQMTQSPSSLSASVGDRVTITCQASQDISNYLNWYQQKPGKAPKLLIYDASNLETGVPSRFSGSGSGTDFTFTISSLQPEDIATYYC-------------
12a21        ......................N...G.G.GSS.....K...R.....VHG....QR..........FH.T..L........D.V...F.AVFQWFGPGTKVDI
min12a21     .........................G.G.GSS...................................FH.T................F.AVFQWFGPGTKVDI
N6           Y.HV........V.I......N..T..GVGSD.H...H...R......HHT.SV.D..........FH.S.NL...D..AD.......QVLQFFGRGSRLHI

VK1-5*01     DIQMTQSPSTLSASVGDRVTITCRASQSISSWLAWYQQKPGKAPKLLIYKASSLESGVPSRFSGSGSGTEFTLTISSLQPDDFATYYC-----------
PCIN63-71I1  A.R.....A...........L....G.G.G.D.G..............H...N.IN..........FH.......N.............QVFEWFGPGTK

VL2-11*01 QSALTQPRSVSGSPGQSVTISCTGTSSDVGGYNYVSWYQQHPGKAPKLMIYDVSKRPSGVPDRFSGSKSGNTASLTISGLQAEDEADYYC---------------
BG24      ...................N.....A------.SGLG.......R....I..E.NR.....S...................RT......F.SAFEYFGEGTKLTVL
```

Figure S2. Comparisons of IOMA to VH1-2 CD4bs bNAbs, related to Figure 1.

(A) Summary of the number of SHM amino acid substitutions, percent mutation, and improbable mutations (calculated by ARMADiLLO [S1-3]) for IOMA-class and VRC01-class bNAbs. Improbable mutations are defined as <1% probability. [S1-3] For nucleotide sequences that were not publicly available for analysis, mprobable mutations are shown as “N/A.” (B) Results of a baculovirus-based polyreactivity ELISA-based assay [S4] evaluating non-specific binding of bovine serum albumin (BSA), a panel of control HIV-1 IgG bNAbs, and IOMA mutant IgG bNAbs. Data are represented as mean RLU ± SEM from four independent replicates (n = 4). (C) Sequence alignment of CD4bs bNAb VH/VL sequences. CDRH regions (as defined by

Kabat numbering) are highlighted in red for CDRH1, blue for CDRH2, and green for CDRH3. CDRL regions (as defined by Kabat numbering) are highlighted in pink for CDRL1, light blue for CDRL2, and light green for CDRL3.

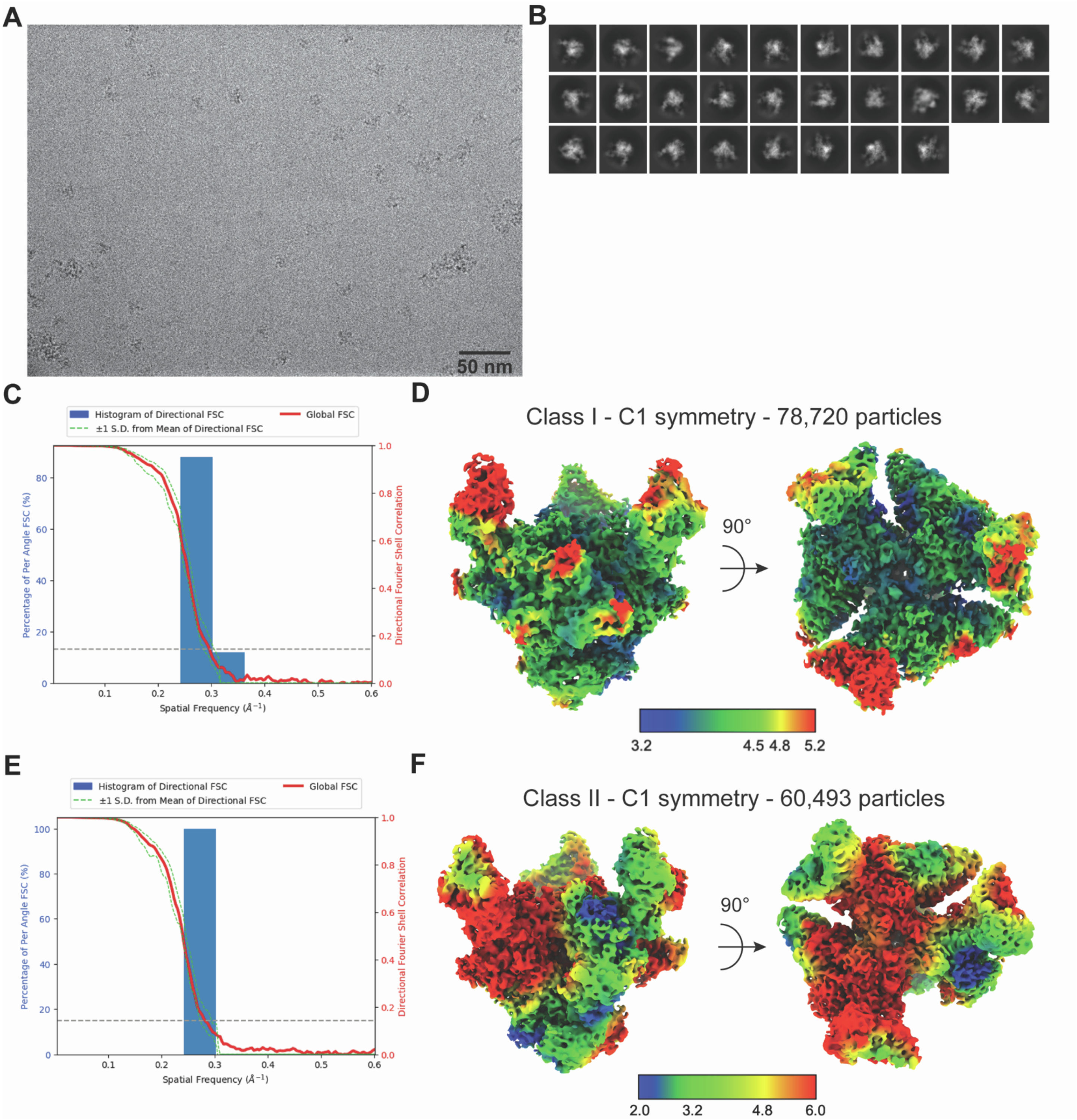

Figure S3. Cryo-EM data processing and validation for IOMAmin5-BG505-10-1074 complexes, related to Figure 3.

(A) Representative micrograph and (B) cryo-EM 2D class averages for the IOMAmin5- BG505-10-1074 cryo-EM structures. For this dataset, two classes were resolved: class I with three IOMAmin5 Fabs bound to BG505 and class II with two IOMAmin5 Fabs bound to BG505. (C) Gold-standard Fourier shell correlation (FSC) plot

and (D) local resolution map for IOMAmin5-BG505-10-1074 class I. (E) Gold-standard FSC plot and (F) local resolution map for IOMAmin5-BG505-10-1074 class II.

Table S1: Cryo-EM data collection, refinement and validation statistics, related to Figure 3.

	**IOMAmin5-10-1074-BG505 Class I** (EMDB-48059) (PDB 9EHL)	**IOMAmin5-10-1074-BG505 Class II** (EMDB-48060) (PDB 9EHM)
**Data Collection and Processing**		
Microscope	Titan Krios	Titan Krios
Camera	Gatan K3	Gatan K3
Magnification	105,000	105,000
Voltage (keV)	300	300
Exposure (e-/Å2)	60	60
Pixel size (Å)	0.4327	0.4327
Defocus Range (um)	-1 to -3	-1 to -3
Initial Particle Image (no.)	860,116	860,116
Final Particle Image (no.)	78,720	60,493
Symmetry Imposed	C1	C1
Map Resolution (Å)	3.9	4.2
FSC Threshold	0.143	0.143
**Refinement**		
Initial Model Used	PDB 5T3X	PDB 5T3X
Model Resolution (Å)	3.9	4.2
FSC Threshold	0.143	0.143
Model composition		
Non-hydrogen atoms	26,146	23,943
Protein residues	3,165	2,898
Ligands	113	99
Average B-factors (Å2)		
Protein	95	104
Ligands	80	89
R.m.s. deviations		
Bond length (Å)	0.006	0.007
Bond angles (º)	1.1	1.1
Validation		
MolProbity score	2.0	2.2
Clashscore	12.4	18.7
Rotamer outliers	0.74	0.72
Ramachandran plot		
Favored (%)	93.2	93.6
Allowed (%)	6.8	6.4
Outliers (%)	0	0

Supplemental References

[S1] Ralph, D.K., and Matsen, F.A. (2019). Per-sample immunoglobulin germline inference from B cell receptor deep sequencing data. PLOS Comput. Biol. 15, e1007133. https://doi.org/10.1371/journal.pcbi.1007133.

[S2] Wiehe, K., Bradley, T., Meyerhoff, R.R., Hart, C., Williams, W.B., Easterhoff, D., Faison, W.J., Kepler, T.B., Saunders, K.O., Alam, S.M., et al. (2018). Functional Relevance of Improbable Antibody Mutations for HIV Broadly Neutralizing Antibody Development. Cell Host Microbe 23, 759-765.e6. https://doi.org/10.1016/j.chom.2018.04.018.

[S3] Martin Beem, J.S., Venkatayogi, S., Haynes, B.F., and Wiehe, K. (2023). ARMADiLLO: a web server for analyzing antibody mutation probabilities. Nucleic Acids Res. 51, W51–W56. https://doi.org/10.1093/nar/gkad398.

[S4] Hötzel, I., Theil, F.-P., Bernstein, L.J., Prabhu, S., Deng, R., Quintana, L., Lutman, J., Sibia, R., Chan, P., Bumbaca, D., et al. (2012). A strategy for risk mitigation of antibodies with fast clearance. mAbs 4, 753–760. https://doi.org/10.4161/mabs.22189.
